# Melflufen in Multiple Myeloma: Clinical Limitations, Biological Rationale, and Future Perspectives

**DOI:** 10.3390/cancers18101551

**Published:** 2026-05-11

**Authors:** Matteo Garibotto, Debora Soncini, Roberto Massimo Lemoli, Antonia Cagnetta, Michele Cea

**Affiliations:** 1 Clinic of Hematology, IRCCS Azienda Ospedaliera Metropolitana, Ospedale Policlinico San Martino, 16132 Genoa, Italy; 2 Clinic of Hematology, Department of Internal Medicine and Medical Specialties (DiMI), University of Genoa, 16132 Genoa, Italy

**Keywords:** multiple myeloma, melflufen, peptide–drug conjugate, aminopeptidases, precision medicine

## Abstract

Despite transformative therapeutic advances, multiple myeloma (MM) remains an incurable malignancy characterized by sequential relapses and progressive treatment resistance. Patients with heavily pretreated relapsed or refractory MM continue to face limited therapeutic options and poor outcomes. Melflufen (melphalan flufenamide) is a peptide–drug conjugate that enhances intracellular delivery of alkylating agents via aminopeptidase-mediated activation. Early clinical studies demonstrated encouraging activity in advanced MM, leading to accelerated approval by the U.S. Food and Drug Administration in 2021. However, results from the phase III OCEAN trial raised concerns regarding overall survival, ultimately resulting in withdrawal of the drug from the U.S. market. In this review, we examine the biological rationale, clinical development, and regulatory trajectory of melflufen, and critically reassess its role within the evolving therapeutic landscape of MM. While the negative survival findings observed in the phase III OCEAN trial substantially limited the clinical development of melflufen, the biological principles underlying enzyme-targeted intracellular drug delivery may still provide relevant insights for biomarker-driven therapeutic strategies and future peptide–drug conjugate development in MM.

## Simple Summary

Multiple Myeloma is a blood cancer characterized by recurrent relapses, with patients refractory to multiple drug classes facing dismal outcomes and limited therapeutic options. Melflufen, a peptide–drug conjugate, exploits increased aminopeptidase activity in myeloma cells to achieve targeted intracellular delivery of the alkylating agent melphalan. Its high lipophilicity enables rapid cellular uptake, followed by enzymatic cleavage and accumulation of cytotoxic metabolites within tumor cells. Despite its approval in Europe for patients with relapsed or refractory Multiple Myeloma, melflufen was withdrawn from the United States market after results from a randomized phase III trial raised concerns regarding inferior overall survival in specific patient subgroups. In this review, we summarize the available biological evidence, clinical development, and current regulatory landscape of melflufen. We also explore its potential positioning within contemporary treatment algorithms. Importantly, the clinical trajectory of melflufen highlighted the critical need for biologically, informed patient selection strategies and reinforces the shift toward precision medicine approaches in Multiple Myeloma.

## 1. Introduction

Multiple myeloma (MM) is the second most common hematologic malignancy and is characterized by the clonal proliferation of malignant plasma cells within the bone marrow, leading to the production of monoclonal immunoglobulins [1,2,3]. Genetic alterations, metabolic dysregulation, and complex interactions with the bone marrow microenvironment contribute to disease progression and to a wide spectrum of clinical manifestations, including bone lesions, hypercalcemia, anemia, immune dysfunction, and renal impairment [4]. According to data from the Global Cancer Observatory (GLOBOCAN), more than 187,000 new cases of MM were diagnosed worldwide in 2022 [5].

Over the past two decades, the therapeutic landscape of MM has undergone profound transformation, resulting in substantial improvements in patient outcomes. Five-year overall survival (OS) has increased from approximately 30% in the 1990s to over 60% in recent years [6]. These advances are largely attributable to the introduction of several novel therapeutic classes, including proteasome inhibitors (PIs), immunomodulatory drugs (IMiDs), monoclonal antibodies, antibody–drug conjugates (ADCs), T-cell-redirecting bispecific antibodies, and chimeric antigen receptor (CAR) T-cell therapies [7]. Combination regimens incorporating these agents, often administered as triplets or quadruplets, now represent the standard of care in both newly diagnosed and relapsed disease settings [8,9].

**Figure 1:**
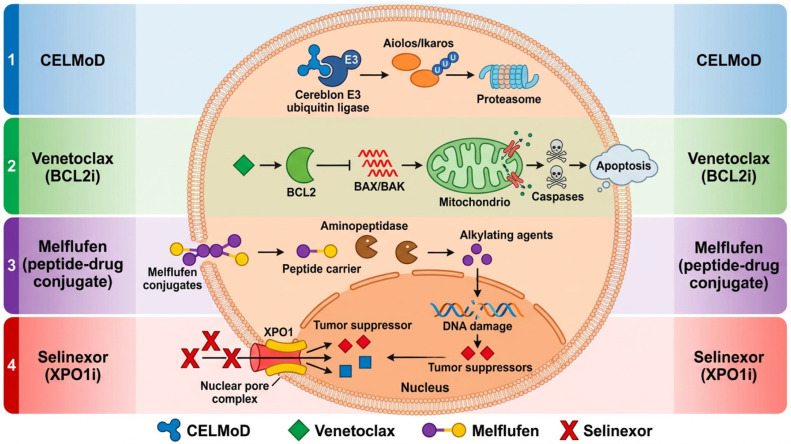
Mechanisms of action of selected therapeutic classes in MM. This schematic summarizes the intracellular mechanisms of action of representative non-T-cell-redirecting and targeted agents used in MM. Cereblon E3 ligase modulators (CELMoDs) induce ubiquitination and degradation of transcription factors such as Aiolos and Ikaros. Venetoclax inhibits BCL2, restoring apoptotic signaling through activation of BAX/BAK. Melflufen, a peptide–drug conjugate, undergoes aminopeptidase-mediated intracellular activation, leading to accumulation of alkylating agents and DNA damage. Selinexor inhibits exportin 1 (XPO1), promoting nuclear retention of tumor suppressor proteins. These agents exemplify mechanistically distinct, non-immune-based therapeutic strategies within the evolving MM landscape.

Despite these advances, MM remains an incurable disease characterized by recurrent relapses and the progressive development of drug resistance [10]. Patients who become refractory to multiple therapeutic classes, particularly those with triple-class refractory disease (i.e., refractory to PIs, IMiDs, and anti-CD38 monoclonal antibodies), represent a difficult-to-treat population with limited therapeutic options and poor prognosis [11,12,13,14]. This unmet clinical need has driven the development of novel therapeutic strategies targeting previously unexploited biological vulnerabilities of MM cells. These include selective inhibitors of nuclear export (SINE), such as selinexor; BCL2 inhibitors, such as venetoclax; cereblon E3 ligase modulators (CELMoDs); antibody–drug conjugates targeting B-cell maturation antigen (BCMA), such as belantamab mafodotin; and peptide–drug conjugates, such as melflufen, designed to enhance the intracellular delivery of cytotoxic agents [15,16,17,18,19] (Figure 1). However, the clinical trajectory of melflufen raises a broader question: how should therapies with discordant efficacy signals be interpreted in the era of rapidly evolving treatment landscapes? In this review, we critically reassess the clinical and biological trajectory of melflufen and discuss whether the mechanistic principles underlying enzyme-targeted drug delivery may still retain translational relevance despite the negative clinical outcomes observed in OCEAN.

## 2. Mechanism of Action of Melflufen

Melphalan, first synthesized in 1953, exerts its cytotoxic activity by alkylating DNA and inducing interstrand cross-links, thereby inhibiting DNA and RNA synthesis and ultimately triggering apoptosis [20]. It has been used in the treatment of MM since the late 1950s and remains a key component of high-dose conditioning regimens for autologous stem cell transplantation (ASCT) [21]. Building on the well-established clinical activity of melphalan, melflufen was developed as a peptidase-potentiated alkylating agent designed to enhance intracellular drug delivery and improve tumor selectivity [22].

**Figure 2:**
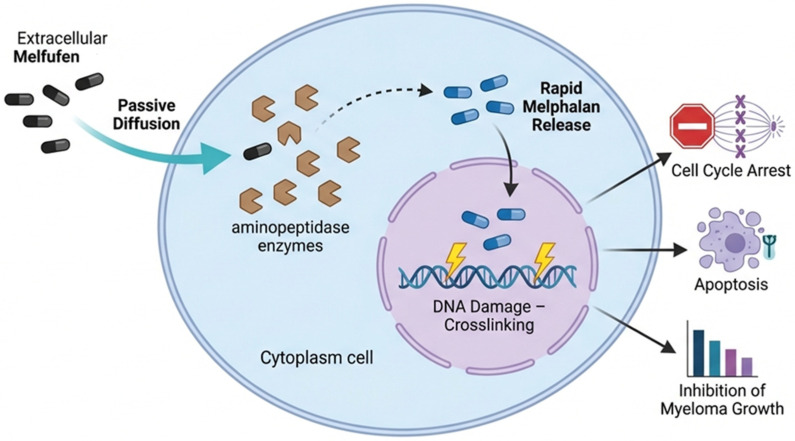
Mechanism of action of melflufen in MM cells. Melflufen, a highly lipophilic peptide–drug conjugate, rapidly diffuses across the plasma membrane of malignant plasma cells. Once internalized, it undergoes enzymatic cleavage by intracellular aminopeptidases and esterases, releasing hydrophilic alkylating metabolites, including melphalan. Due to their limited membrane permeability, these metabolites are retained within the cell, resulting in intracellular accumulation of cytotoxic payload. This leads to extensive DNA damage, including interstrand cross-linking in nuclear and mitochondrial DNA, ultimately triggering cell cycle arrest and apoptosis. This mechanism exemplifies enzyme-targeted intracellular drug delivery resulting in selective intracellular retention of alkylating agents within tumor cells.

Melflufen is a highly lipophilic peptide–drug conjugate composed of a melphalan-derived alkylating moiety linked to a peptide carrier (p-L-fluorophenylalanine ethyl ester), which facilitates rapid passive diffusion across cellular membranes [23,24] (Figure 2). Once inside the cell, melflufen undergoes rapid enzymatic hydrolysis mediated by intracellular peptidases and esterases, leading to the release of hydrophilic alkylating metabolites, including melphalan and desethyl-melflufen [22,25]. Because these metabolites exhibit limited membrane permeability, they become trapped within the cell, resulting in the intracellular accumulation of cytotoxic alkylating compounds [22,26].

The preferential activation of melflufen within malignant plasma cells is largely driven by aminopeptidases, a family of proteolytic enzymes that catalyze the hydrolysis of N-terminal amino acids from peptides [20]. Aminopeptidases play a key role in protein turnover and are frequently overexpressed in malignant cells, including MM. Elevated expression of several aminopeptidases such as aminopeptidase N (ANPEP/CD13), LAP3, and DPP7 has been associated with disease progression and poor prognosis, suggesting that these enzymes may represent a therapeutic vulnerability [27,28]. Following intracellular activation, melflufen-derived metabolites induce extensive DNA damage, including cross-links affecting both nuclear and mitochondrial DNA, ultimately leading to apoptosis. In addition, melflufen disrupts mitochondrial membrane potential, further contributing to tumor cell death [25,29,30]. Importantly, preclinical studies indicate that the cytotoxic activity of melflufen is largely independent of TP53 mutational status, a feature associated with poor prognosis and resistance to therapy in MM [25,26,31]. Moreover, melflufen appears less susceptible to multidrug resistance mechanisms mediated by multidrug resistance protein 1 (MDR1/ABCB1), which contribute to resistance to several anti-myeloma agents [32,33,34].

Taken together, these properties allow melflufen to achieve enhanced intracellular retention of alkylating metabolites following aminopeptidase-mediated activation. This pharmacologic strategy was developed to increase intracellular delivery of alkylating agents in MM cells while exploiting tumor-associated peptidase activity [35].

## 3. Preclinical Development

Preclinical studies demonstrated that melflufen exhibits potent cytotoxic activity in MM cell lines as well as in primary patient-derived myeloma cells. Compared with melphalan, melflufen achieves significantly higher intracellular accumulation of alkylating metabolites, resulting in enhanced DNA damage and apoptosis [36,37]. Consistent with its mechanism of action, melflufen retains activity in several models of drug-resistant disease. In vitro studies have demonstrated cytotoxic effects in both melphalan- and bortezomib-resistant myeloma cell lines, with significantly lower IC_50_ values compared with melphalan [26]. Beyond its direct cytotoxic effects, melflufen may also modulate the bone marrow (BM) microenvironment. Preclinical studies have shown that the drug inhibits the proliferation of BM-derived mesenchymal stromal cells, which are known to support MM cell growth and contribute to drug resistance [35,38,39]. In addition, melflufen has demonstrated anti-angiogenic activity and has been shown to inhibit monocyte proliferation, suggesting potential effects on osteoclastogenesis and bone remodeling [40,41,42,43,44]. Evidence from other malignancies further supports the tumor-selective activity of melflufen. Studies in breast cancer models have shown that malignant cells are more sensitive to melflufen than normal epithelial cells, while experiments in primary acute myeloid leukemia samples demonstrated marked cytotoxicity in leukemic cells, with relatively lower sensitivity observed in peripheral blood mononuclear cells [38,45]. Overall, these findings provide a strong biological rationale for the clinical development of melflufen in patients with relapsed or refractory MM.

## 4. Clinical Development

The clinical development of melflufen has primarily focused on patients with relapsed or refractory multiple myeloma (RRMM). Early-phase studies demonstrated encouraging anti-myeloma activity with a manageable safety profile, supporting further evaluation in larger trials.

The first clinical assessment of melflufen in RRMM was the multicenter phase I/II O-12-M1 study, which evaluated melflufen in combination with dexamethasone in patients who had received at least two prior lines of therapy, including lenalidomide or bortezomib, and were refractory to their most recent treatment [46,47]. Eligible patients had measurable disease and an Eastern Cooperative Oncology Group (ECOG) performance status ≤2. The phase I portion established the maximum tolerated dose, while the phase II portion assessed efficacy and safety. In the phase II cohort, 45 patients received melflufen 40 mg intravenously every 28 days plus weekly dexamethasone. The median age was 66 years, and patients had received a median of four prior lines of therapy. The population was enriched for high-risk features: 67% were double-refractory, 58% had undergone prior ASCT, and 44% had high-risk cytogenetics. After a median follow-up of 27.9 months, the overall response rate (ORR) was 31%, including 20% partial responses and 11% very good partial responses. The clinical benefit rate was 49%, with a median duration of response (DOR) of 8.4 months. Median progression-free survival (PFS), overall survival (OS), and time to next treatment were 5.7, 20.7, and 7.9 months, respectively. Notably, responses were observed in patients with melphalan-resistant disease, and high-risk cytogenetics did not appear to adversely impact OS. Toxicity was predominantly hematologic, with thrombocytopenia, neutropenia, and anemia as the most common adverse events. Non-hematologic toxicities were generally manageable and included fatigue, pyrexia, gastrointestinal symptoms, and bone pain. Serious adverse events occurred in 38% of patients, most frequently infections and febrile neutropenia.

These findings were further explored in the phase II HORIZON (OP-106) study, a single-arm trial enrolling 157 heavily pretreated RRMM patients refractory to pomalidomide and/or an anti-CD38 monoclonal antibody [48,49]. Patients had received a median of five prior lines of therapy, and a substantial proportion had triple-class refractory disease. After a median follow-up of 14 months, the ORR was 29%, including 11% very good partial responses and 18% partial responses. Median DOR, PFS, and OS were 5.5, 4.2, and 11.6 months, respectively. Importantly, clinically meaningful activity was maintained in the triple-class refractory subgroup (ORR 26%).

The phase III OCEAN trial subsequently compared melflufen plus dexamethasone with pomalidomide plus dexamethasone in patients with RRMM refractory to lenalidomide after two to four prior lines of therapy [50,51,52]. Among 495 randomized patients, melflufen improved the primary endpoint of PFS (6.8 vs. 4.9 months) and achieved a numerically higher ORR (33% vs. 27%). However, overall survival favored the pomalidomide arm (25.0 vs. 19.8 months). Subgroup analyses suggested a differential effect according to prior transplant history: patients without prior ASCT or relapsing more than 36 months after transplantation appeared to benefit from melflufen, whereas those with early post-transplant relapse experienced inferior outcomes [53,54].

The discordance between PFS and OS in the OCEAN trial warrants careful interpretation and raises important biological and methodological questions. Importantly, the observed overall survival detriment cannot be dismissed as a purely methodological artifact and represents a clinically meaningful safety concern that substantially limits enthusiasm for broad clinical implementation of melflufen. At the same time, differences in post-progression therapies may have contributed to survival outcomes, particularly within a rapidly evolving therapeutic landscape characterized by heterogeneous access to subsequent treatments. In addition, the negative effect observed in specific subgroups, notably patients with early relapse after ASCT, suggests that prior treatment exposure and disease biology may significantly influence the therapeutic index of melflufen. Cumulative hematologic toxicity may also have affected treatment delivery or predisposed to complications impacting long-term survival. Trial design factors, including potential imbalances in patient characteristics and subsequent treatment strategies, may have further contributed to the observed survival outcomes. More broadly, these findings highlight the limitations of PFS as a surrogate endpoint in heavily pretreated MM populations and underscore the need for refined patient selection and more nuanced interpretation of efficacy outcomes.

These results led to a complex regulatory trajectory. In the United States, melflufen initially received accelerated approval in 2021 for patients who had received at least four prior lines of therapy but was subsequently withdrawn following the OCEAN findings, with formal confirmation by the U.S. Food and Drug Administration [24,55]. In contrast, the European Medicines Agency granted conditional approval in 2022 for a more selected population, namely patients with at least three prior lines of therapy who had not undergone prior ASCT or had relapsed more than three years after transplantation [56,57].

Melflufen has also been investigated in combination strategies aimed at improving efficacy. The phase I/IIa ANCHOR study investigated melflufen plus dexamethasone in combination with daratumumab or bortezomib in patients who had received one to four prior lines of therapy [58]. In the daratumumab cohort (*n* = 33), the ORR was 73%, with a median PFS of 12.9 months and a median OS of 26.1 months. In the bortezomib cohort (*n* = 23), the ORR was 78%, with a median PFS of 14.7 months, although OS data remained immature. Toxicity was consistent with previous studies and predominantly hematologic. However, given the limited sample size and non-randomized design, these results should be interpreted with caution. The phase III LIGHTHOUSE trial evaluated melflufen in combination with daratumumab and dexamethasone versus daratumumab alone in patients with RRMM who had received at least three prior lines of therapy or were refractory to both an immunomodulatory drug and a proteasome inhibitor [59]. Although the triplet regimen demonstrated improved response rates (ORR 59% vs. 30%), the study was prematurely terminated following a partial clinical hold requested by the U.S. Food and Drug Administration during the regulatory reassessment of melflufen (Table 1).

## 5. Real-World Evidence and Special Populations

Evidence on the real-world use of melflufen in RRMM patients remains limited but provides important insights into its activity in heavily pretreated populations that are often underrepresented in clinical trials. One of the earliest retrospective analyses, conducted at the Dana-Farber Cancer Institute, included 12 patients who had received a median of 5.5 prior lines of therapy [60]. Treatment with melflufen plus dexamethasone achieved an ORR of 55%, including complete responses in 27% of patients. The median duration of response was 21.3 weeks, and the median time to treatment failure was 29.7 weeks. Adverse events were predominantly hematologic and generally manageable with supportive measures such as transfusions and growth factor support. Infections were relatively uncommon, and no cases of mucositis, alopecia, or secondary malignancies were reported.

Additional real-world experiences from small European retrospective cohorts have yielded broadly consistent results. A Spanish series including seven heavily pretreated patients reported an ORR of 57% [61], while a multicenter cohort of 19 patients demonstrated an ORR of 26% and a median progression-free survival (PFS) of 4.8 months despite a high prevalence of triple- and penta-class refractory disease [62]. Similar outcomes have been observed in Italian cohorts, with response rates ranging from 37.5% to 47% in advanced disease settings [63,64]. Across studies, hematologic toxicity, particularly thrombocytopenia, neutropenia, and anemia, represented the predominant adverse event profile and frequently required dose modifications and supportive care measures. Although these retrospective observations suggest potential activity in selected heavily pretreated patients, their interpretation remains substantially limited by small cohort sizes, selection bias, heterogeneous patient populations, and the absence of randomized comparisons. Notably, several cohorts included patients previously exposed to T-cell-redirecting therapies or BCMA-targeted agents, raising the possibility that melflufen may retain activity in immunotherapy-exposed disease. Beyond advanced RRMM, emerging evidence supports the feasibility of melflufen in specific clinical subgroups. The phase I/II BRIDGE (OP-107) study evaluated its pharmacokinetics and safety in patients with renal impairment [65]. Although systemic exposure to melphalan increased with decreasing estimated glomerular filtration rate (eGFR), no clinically meaningful differences in toxicity were observed, suggesting that melflufen can be administered in patients with moderate renal dysfunction without dose adjustment.

Taken together, these observations suggest potential activity in heavily pretreated and immunotherapy-exposed patients. However, these findings should be interpreted cautiously given the retrospective design, small sample sizes, and heterogeneity of patient populations. These limitations preclude definitive conclusions and highlight the need for prospective studies and registry-based analyses to better define the effectiveness, safety, and optimal positioning of melflufen in routine clinical practice.

## 6. Positioning of Melflufen in the Evolving Treatment Landscape of MM

In the current immunotherapy-driven era, the clinical positioning of melflufen should move beyond a purely descriptive framework toward a more decision-oriented approach grounded in mechanism, patient characteristics, and treatment sequencing [12,66,67]. Rather than competing directly with T-cell-redirecting therapies, melflufen may be conceptualized as a complementary, non-immune-based strategy in selected clinical contexts. Compared with CAR T-cell therapies and bispecific antibodies, melflufen does not rely on immune competence or antigen expression, which may represent an advantage in patients with T-cell exhaustion or antigen escape [68,69,70,71]. However, its overall efficacy appears more modest, suggesting that melflufen may be more appropriately considered a complementary rather than competing therapeutic strategy. Despite the limitations emerging from OCEAN, selected clinical scenarios have been proposed in which melflufen could still retain relevance as a non-immune-based therapeutic option [72]. First, melflufen may serve as a bridging strategy in patients awaiting access to CAR T-cell therapies or bispecific antibodies. In this setting, its rapid cytotoxic activity and independence from immune effector function may allow for disease control without impairing subsequent immunotherapy, particularly in patients with high disease burden requiring prompt cytoreduction. Second, melflufen may represent a therapeutic option in patients relapsing after T-cell-redirecting therapies. Post-immunotherapy relapse is increasingly characterized by antigen escape, T-cell exhaustion, and limited responsiveness to further immune-based interventions. Given its mechanism of action, which is independent of both antigen expression and T-cell fitness, melflufen may retain activity in this setting and provide a mechanistically orthogonal approach. Third, melflufen may be relevant in patients who are not candidates for cellular or T-cell-redirecting therapies due to advanced age, comorbidities, frailty, or limited access to specialized centers. In this population, there remains a need for additional non-immune-based therapeutic options [49,52].

Importantly, available data suggest that patient selection is a key determinant of benefit–risk balance. Subgroup analyses from the OCEAN trial indicate that prior ASCT and timing of relapse significantly influence outcomes, with improved results observed in patients without prior ASCT or with late relapse after transplantation [54]. Conversely, inferior outcomes in early post-transplant relapse highlight the need for careful selection. In clinical practice, the selection of melflufen should be weighed against available alternatives, including CELMoDs, bispecific antibodies, and CAR T-cell therapies, considering treatment availability, prior exposure, disease kinetics, and patient fitness [12,73,74,75,76].

**Figure 3:**
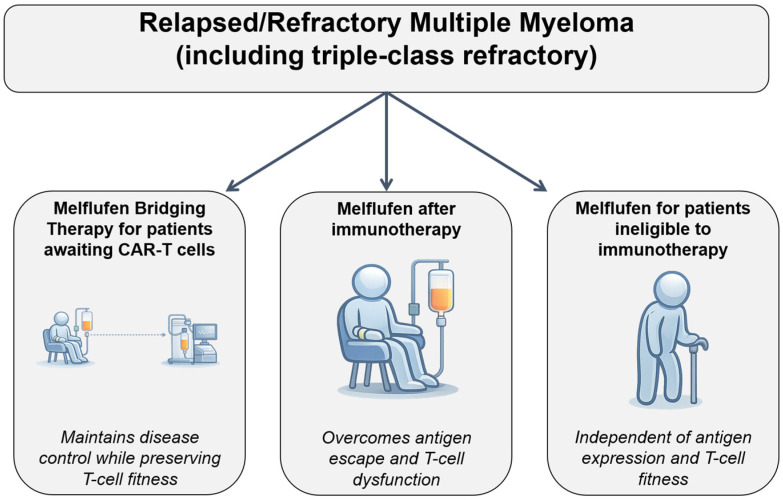
Proposed clinical positioning of melflufen in RRMM pts. In the current immunotherapy-driven landscape, melflufen represents a mechanistically distinct, non-T-cell-redirecting therapeutic strategy based on enzyme-targeted intracellular delivery of alkylating agents. This schematic illustrates three potential clinical scenarios for its use: (1) bridging therapy in patients awaiting access to CAR T-cell or bispecific antibody treatments; (2) post-immunotherapy relapse, where resistance is often driven by antigen escape or T-cell dysfunction; and (3) patients ineligible for T-cell-based therapies due to frailty, comorbidities, or limited access to specialized centers. Dashed arrows indicate that optimal sequencing remains undefined and should be guided by prior treatments, disease biology, and patient-specific factors.

Taken together, these considerations support a more restrictive, biology- and context-driven use of melflufen. Ideal candidates may include patients with heavily pretreated disease requiring rapid disease control, those relapsing after immunotherapy, and individuals ineligible for T-cell-redirecting approaches (Figure 3). In contrast, patients with early relapses after ASCT or those with highly effective alternative options available may derive limited benefit. Future efforts should aim to refine this positioning through prospective studies and biomarker-driven strategies, enabling a more precise integration of melflufen within an increasingly complex therapeutic landscape. To contextualize the clinical positioning of melflufen within the current therapeutic landscape, a comparison with selected available treatment options is summarized in Table 2. However, cross-trial comparisons should be interpreted cautiously given differences in patient populations, prior therapies, and study design.

## 7. Safety Profile

The safety profile of melflufen is predominantly characterized by hematologic toxicity [72]. The most frequently reported adverse events include thrombocytopenia, neutropenia, and anemia, which are generally manageable through dose modifications, treatment delays, and supportive measures such as transfusions or growth factor support. Non-hematologic adverse events occur less frequently and primarily include infections, fatigue, and gastrointestinal symptoms. Nevertheless, in a substantial proportion of patients these toxicities were dose-limiting and frequently required treatment interruptions, dose reductions, transfusion support, or growth factor administration [58]. However, the overall survival findings reported in the phase III OCEAN trial raised concerns regarding potential long-term risks in specific patient subgroups, particularly in relation to prior transplant exposure. These observations underscore the importance of careful patient selection and the need for continued evaluation of safety outcomes in both clinical trials and real-world settings.

## 8. Future Perspectives

Despite the regulatory challenges encountered during its clinical development, melflufen represents a compelling proof of concept for enzyme-targeted intracellular drug delivery. By exploiting tumor-associated aminopeptidase activity to enhance intracellular accumulation of cytotoxic agents, this approach represents a mechanistically distinct strategy for selective intracellular delivery of alkylating agents. More broadly, it highlights the potential of peptide–drug conjugates to achieve tumor-selective intracellular activation of cytotoxic agents through exploitation of cancer-associated enzymatic activity. Future research should extend beyond melflufen itself and focus on the development of next-generation peptide–drug conjugates that exploit tumor-specific enzymatic vulnerabilities.

In parallel, a deeper understanding of the biological determinants of response and toxicity will be essential to optimize the clinical application of these agents within an increasingly complex therapeutic landscape. A critical unmet need is the identification of predictive biomarkers to guide patient selection [77]. Given its mechanism of action, aminopeptidase expression and activity represent biologically plausible candidates. Candidate biomarkers may include CD13 (ANPEP) expression levels, which could be assessed by immunohistochemistry or transcriptomic profiling to stratify patients prior to treatment [27]. Preclinical data suggest that higher aminopeptidase activity enhances intracellular activation of melflufen and cytotoxic efficacy; however, these approaches remain exploratory and lack prospective validation.

At present, no validated biomarkers are available for routine clinical use, and aminopeptidase-based stratification remains investigational. Future studies should aim to identify the most relevant aminopeptidases, standardize methods for their assessment (including immunohistochemistry, transcriptomic profiling, and functional enzymatic assays), and define clinically meaningful thresholds. Importantly, biomarker analyses should be prospectively integrated into clinical trials to enable validation of their predictive value. Ultimately, a biomarker-driven approach may allow for more precise patient selection, improving the benefit–risk balance of melflufen and supporting its integration into a personalized, mechanism-based treatment strategy in MM.

## 9. Current Limitations and Unresolved Questions

Despite encouraging mechanistic rationale and early clinical activity, several major limitations continue to prevent a clear definition of the clinical role of melflufen in MM. Most importantly, the negative overall survival signal observed in OCEAN remains incompletely understood and represents a major unresolved safety concern. In addition, no validated biomarkers are currently available to identify patients more likely to benefit from aminopeptidase-targeted drug delivery strategies. Interpretation of currently available real-world evidence is further limited by retrospective study design, small cohort sizes, and substantial clinical heterogeneity. Finally, the rapidly evolving therapeutic landscape of MM, particularly following the introduction of highly active T-cell-redirecting therapies, further complicates the positioning of alkylating-based approaches. Addressing these challenges will require prospective biomarker-driven studies, improved understanding of treatment-related toxicity, and more refined patient selection strategies.

## 10. Conclusions

Melflufen represents a therapeutic strategy based on enzyme-targeted intracellular delivery of alkylating agents, developed to enhance selective drug accumulation in MM cells. Early clinical studies demonstrated encouraging activity in heavily pretreated patients, leading to accelerated regulatory approval. However, the results of the phase III OCEAN trial raised important concerns, particularly regarding overall survival in specific patient subgroups, prompting a substantial reassessment of its clinical role. Despite these limitations, the clinical development of melflufen provided important insights into enzyme-targeted drug delivery and biomarker-guided therapeutic positioning in MM. These concepts may inform the future development of next-generation peptide–drug conjugates and other biology-driven therapeutic strategies.

In the current immunotherapy era, the role of melflufen is likely to be confined to selected clinical scenarios, including patients with limited therapeutic options, those ineligible for cellular therapies, and individuals relapsing after T-cell-redirecting approaches. In these settings, its mechanism of action may offer a complementary, non-immune-based strategy.

Ultimately, the trajectory of melflufen highlights a broader principle for modern myeloma therapy: effective treatment increasingly depends on the rational integration of mechanistically distinct approaches guided by disease biology and patient-specific factors. Future progress will require precise patient selection, biomarker-driven strategies, and optimized treatment sequencing to fully exploit the potential of emerging therapeutic platforms.

## Figures and Tables

**Table 1:** Summary of clinical trials evaluating melflufen in RRMM patients.

Trial (Phase)	Treatment	Patients	Median Follow-Up (Months)	ORR (%)	mPFS (Months)	mOS (Months)	Ref.
O-12-M1 (I–II)	Melflufen + DEX	45	27.9	31	5.7	20.7	[47]
HORIZON (II)	Melflufen + DEX ^a^	157	14	29	4.2	11.6	[49]
OCEAN (III)	Melflufen + DEX ^a^ vs. Pom + DEX ^a^	246 vs. 249	15.5 vs. 16.3	33 vs. 27	6.8 vs. 4.9	19.8 ^b^ vs. 25 ^c^	[51]
ANCHOR (I–II)	Melflufen + Dara ^d^ + DEX ^a^ vs. Melflufen + Bz + DEX ^a^	33 vs. 23	30.2 vs. 21.0	73 vs. 78	12.9 vs. 14.7	26.1 ^e^ vs. NR ^f^	[58]
LIGHTHOUSE (III)	Melflufen + Dara ^g^ + DEX ^a^ vs. Dara ^g, h^ + DEX	27 vs. 27	7.1 vs. 6.6	59 vs. 30	NR vs. 4.9	11 vs. NR	[59]

DEX: dexamethasone; Bz: bortezomib; Dara, daratumumab; NR: not reached. ^a^ DEX was reduced to 20 mg in patients with age ≥ 75 years. ^b^ At a median follow-up of 19.8 months. ^c^ At a median follow-up of 18.6 months. ^d^ Daratumumab 16 mg/kg was administered intravenously on D 2, 8, 15 and 22 in cycle 1, weekly on cycle 2, every other week on cycles 3–6 and on day 1 from cycle 7 onward. ^e^ At a median follow-up of 32.8 months. ^f^ At a median follow-up of 17.6 months OS data were considered immature. ^g^ Daratumumab 1800 mg was administered weekly for the first 2 cycles, every other week in cycles 3–6 and only on day 1 in cycle 7 onward. ^h^ Patients in the daratumumab group received dexamethasone 20 mg (or an equivalent glucocorticoid) pre-dose, with an allowed dose reduction to 12 mg from the second injection, and dexamethasone 4 mg orally for two days post-dose.

**Table 2:** Comparative efficacy of selected therapies in RRMM pts.

Therapy	Mechanism	Setting	ORR (%)	mPFS (Months)	mOS (Months)	Key Limitations	Ref.
Melflufen + DEX	Peptide–drug conjugate	RRMM (≥2 prior lines)	33	6.8	19.8	OS detriment in OCEAN; hematological toxicity	[51]
Pomalidomide + DEX	IMiD	RRMM	27	4.9	25.0	Resistance in heavily pretreated patients	[51]
Teclistamab	TCE (BCMA)	RRMM (Triple-class exposed)	63	~11–12	NR	Infections; CRS	[73]
Elranatamab	TCE (BCMA)	RRMM (Triple-class exposed)	61	~12	NR	Infections; CRS	[74]
Ide-cel	CAR-T (BCMA)	RRMM (≥3 prior lines)	~70–75	~8–9	~24	Manufacturing time; CRS; ICANS; relapse	[76]
Cilta-cel	CAR-T (BCMA)	RRMM (≥3 prior lines)	~95–97	~34	NR	Manufacturing time; CRS; ICANS	[75]

RRMM: relapsed/refractory MM pts; TCE: T-cell engagers; CAR-T: chimeric antigen receptor T cells; NR: not reached.

## Data Availability

No new datasets were generated or analyzed in this study.

## Abbreviations

The following abbreviations are used in this manuscript:

ADC	Antibody–drug Conjugate
ASCT	autologous stem cell transplantation
BM	bone marrow
Bz	bortezomib
CAR-T	chimeric antigen receptor T
CELMoDs	cereblon E3 ligase modulators
Dara	Daratumumab
DEX	dexamethasone
DOR	duration of response
MDR1/ABCB1	multidrug resistance protein 1
MM	Multiple Myeloma
NR	Not reached
ORR	overall response rate
OS	Overall Survival
PFS	progression-free survival
PI	Proteasome inhibitor
Pom	Pomalidomide
RRMM	relapsed or refractory multiple myeloma
SINE	selective inhibitors of nuclear export
TCE	T-cell engagers
